# Patient factors to target for elimination of mother-to-child transmission of HIV

**DOI:** 10.1186/1744-8603-10-36

**Published:** 2014-05-15

**Authors:** Coceka N Mnyani, Adonia Simango, Joshua Murphy, Matthew Chersich, James A McIntyre

**Affiliations:** 1 Department of Obstetrics and Gynaecology, and School of Public Health, Faculty of Health Sciences, University of the Witwatersrand, Johannesburg, South Africa; 2 Anova Health Institute, 12 Sherborne Road, Parktown, 2193 Johannesburg, South Africa; 3 Centre for Health Policy, School of Public Health, University of Witwatersrand, Johannesburg, South Africa; 4 Wits Reproductive Health and HIV Research Institute, Faculty of Health Sciences, University of the Witwatersrand, Johannesburg, South Africa; 5 International Centre for Reproductive Health, Department of Obstetrics and Gynaecology, Ghent University, Ghent, Belgium; 6 School of Public Health and Family Medicine, University of Cape Town, Cape Town, South Africa

**Keywords:** Elimination of mother-to-child transmission, HIV and pregnancy, Patient factors

## Abstract

**Background:**

There is great impetus to achieve elimination of mother-to-child transmission of HIV (eMTCT) by 2015, and part of this is to identify factors to target to achieve the goal. This study thus identified key patient factors for MTCT in a high HIV prevalence setting in Johannesburg, South Africa. Between November 2010 and May 2012, we conducted a case–control study among HIV-infected women with HIV-infected (cases) and uninfected (controls) infants diagnosed around six weeks of age as part of routine, early infant diagnosis. Mothers and infants were identified through registers in six healthcare facilities that provide antenatal, postpartum and HIV care. Structured interviews were conducted with a focus on history of HIV infection, antenatal, intrapartum and immediate postpartum management of the mother-infant pair. Patient-related risk factors for MTCT were identified.

**Results:**

A total of 77 women with HIV-infected infants and 154 with –uninfected infants were interviewed. Among HIV-infected cases, 13.0% of the women knew their HIV status prior to conception, and 83.1% reported their pregnancies as unplanned. Antenatal antiretroviral coverage was high in the control group – only 1/154 (0.7%) reported receiving no prophylaxis or treatment compared with 17/74 (22.9%) of cases. In multivariate analysis, key patient-related risks for HIV transmission were: unknown HIV status prior to conception (adjusted odds ratio [AOR] = 6.6; 95% CI = 2.4 – 18.4; *p* < 0.001); accessing antenatal care after 20 weeks gestation (AOR = 4.3; 95% CI = 2.0 – 9.3; *p* < 0.001); less than 12 years of formal education (AOR = 3.4; 95% CI = 1.6 – 7.5; *p* = 0.002); and unplanned pregnancy (AOR = 2.7; 95% CI = 1.2 to 6.3; *p* = 0.022). Mean age at first HIV test was 6.6 weeks (SD = 3.5) for infants who were diagnosed as HIV-infected, and the mean age at antiretroviral treatment initiation was 10.8 weeks (SD = 4.4). HIV-uninfected infants were diagnosed at a mean age of 6.0 weeks (SD = 0.2).

**Conclusions:**

Undiagnosed maternal HIV infection prior to conception, unplanned pregnancies, delays in accessing antenatal care, and low levels of education were the most significant patient risk factors associated with MTCT. While the emphasis has been on increasing availability and coverage of efficacious antiretroviral regimens, and strengthening health systems within eMTCT initiatives, there is a need to also address patient-related factors if we are to achieve eMTCT goals.

## Background

In 2009, UNAIDS, the Joint United Nations Programme on HIV/AIDS, made a global call for the elimination of mother-to-child transmission of HIV (eMTCT) by 2015, and there is great impetus to achieve the target as the deadline approaches [1-3]. Coupled with this was a call for a 50% reduction in HIV-related maternal mortality [1]. Elimination is defined as a 90% reduction in the number of new paediatric HIV infections, or a MTCT rate below 5% [1]. Great strides have been made in the field of prevention of mother-to-child transmission (PMTCT), both globally and in South Africa [3]. Rates of HIV transmission to children have decreased substantially – among the 21 Global Plan priority countries in sub-Saharan Africa, including South Africa, MTCT rates have decreased by around a half or more since 2009 [3]. This decrease is largely attributable to the availability of efficacious antiretroviral regimens at high coverage [3]. However, success of PMTCT initiatives depends on more than the availability of antiretrovirals. The World Health Organization’s (WHO’s) comprehensive strategic approach for PMTCT also includes primary prevention of HIV infection; prevention of unintended pregnancies in HIV-infected women; and a family-centred approach to HIV care [4]. There is also a need to integrate PMTCT into routine maternal, neonatal, child and women’s health services [5]. Several studies have demonstrated the benefits of integration of services, in both the delivery and utilisation of services [5].

Patient and health system factors are critical in determining success of PMTCT programmes and attainment of eMTCT goals [6-9]. While there are effective biomedical interventions for HIV and pregnancy prevention, patients need timely access to care and then to utilise the interventions appropriately. Women who are already pregnant need to access healthcare early and if identified as HIV-infected, be initiated on antiretrovirals timeously and also adhere to therapy during the ante- and postpartum periods. While it is well-recognised that the highest risk of MTCT is among women with undiagnosed and/or untreated HIV infection, and also in known HIV-infected women who either do not start or delay treatment, there are still health system factors that fail to address these [10-13]. Also, antenatal and HIV services remain largely segregated, and there is still failure to prioritise pregnant women for initiation of antiretroviral therapy (ART) [12,13]. Pregnant women may not access antenatal care early; may decline ART; or fail to adhere to antiretroviral regimens. This study thus focused on, and identified key patient-centred risks for MTCT in a high HIV prevalence setting in Johannesburg, South Africa.

## Methods

### Study design

Between November 2010 and May 2011, consecutive mothers with HIV-infected infants who were referred to a paediatric HIV clinic for ART initiation were enrolled (cases). Mothers of uninfected infants (controls), identified through clinic registers, were recruited at five primary care facilities between December 2011 and May 2012. Mothers of both HIV-infected and –uninfected infants were all HIV-infected. The decision to obtain controls was taken after analysis of data on mothers of infected infants showed interesting patterns on patients’ health-seeking behaviour and care accessed during pregnancy. The controls and cases were similar with respect to site of antenatal care, place and date of delivery, and where infant HIV testing was done. Both group of infants were also born in the same period, but controls were recruited slightly later than cases.

Twice as many controls as cases were enrolled and a convenience sample was selected. The initial plan was to enrol more controls, but due to difficulties with recruitment of mothers with uninfected infants and concerns about recall bias from the lengthy period between the cases and controls, the ratio was decreased to 2:1. Of the controls that were contacted, a quarter of them could be reached and were available to be interviewed. There were several reasons why mothers of uninfected infants were unavailable for interview, and these included the women having returned to work and unable to take time off; migration; and contact number unavailable or changed. The 2:1 ratio was deemed adequate for detecting differences between the two groups.

Participants were interviewed using a structured questionnaire and data on the following parameters were collected from the interviews: history of HIV infection – whether preconception HIV testing was done, and whether ART was initiated in those identified as HIV-infected; baseline CD4 cell count; knowledge of partner’s HIV status; whether the pregnancy was planned; previous exposure to PMTCT interventions; antenatal and intrapartum prophylaxis; infant prophylaxis and feeding option; and timing of early infant diagnosis. Additional data were collected on management of infant HIV infection in cases of HIV-infected infants. Infants diagnosed as either HIV-infected or –uninfected, through early infant diagnosis, were identified through facility registers. Interviewers were fluent in the local vernacular languages used in the interviews. Internal audit processes were conducted on the study data, including validation of information collected from interviews against facility records.

### Study setting

Within routine care, all facilities in Soweto, Johannesburg where the women accessed care provided PMTCT services during the antenatal and postnatal periods. HIV testing was routinely offered to all pregnant women, and if HIV-infected, either antiretroviral prophylaxis or lifelong treatment was initiated, depending on a woman’s CD4 cell count. All antenatal clinics provided antiretroviral prophylaxis, while ART initiation for those eligible occurred only at selected sites over the study period. Postnatal testing was offered if a woman’s HIV status was unknown, and also in those who tested HIV negative during pregnancy and did not have a repeat test antenatally. A free six months’ supply of infant formula was given to HIV-infected women who elected to formula feed. All health facilities offered routine early infant diagnosis around six weeks of age, but management of infected infants occurred only at one paediatric HIV clinic at the time of the study.

Over the study period, uptake of HIV testing within the antenatal clinics was 99%, and the HIV prevalence in pregnant women was around 25% (unpublished programme data). MTCT risk around six week was 2.7% in 2010 and 1.1% in 2011. The study period coincided with PMTCT guideline changes (Table 1); most notable was the initiation of zidovudine prophylaxis from an earlier gestational age and an increase in CD4 cell count threshold for ART initiation, from 200 to 350 cells/mm^3^. There was also a transition from zidovudine to nevirapine syrup for infant prophylaxis.

**Table 1:** Summary of key changes in South African PMTCT guidelines

**Date**	**PMTCT guidelines**
**2002 to February 2008**	Only intrapartum and infant sdNVP available for prophylaxis
**2004**	ART became available; CD4 threshold for ART initiation 200 cells/mm^3^
**February 2008**	Maternal AZT (from 28 weeks gestation) and infant AZT, for prophylaxis. One to four weeks of infant AZT, depending on duration of maternal prophylaxis or treatment
**April 2010**	Gestational age for AZT initiation reduced to 14 weeks, CD4 threshold for ART initiation increased to 350 cells/mm^3^, Infant NVP syrup prophylaxis introduced for breastfeeding period – six weeks if preconception initiation of maternal ART; extended to cover breastfeeding period if no maternal ART
**April 2012**	Withdrawal of free infant formula, EFV-based ART regimen approved for pregnant women
**April 2013**	EFV-based FDCs for prophylaxis and treatment introduced

sdNVP single-dose nevirapine; AZT zidovudine; EFV efavirenz; FDCs fixed-dose combinations.

### Data analysis

Data were analysed with Stata® version 12.0 (Stata Corporation, College Station, USA). Continuous data were summarized using means and medians as appropriate. Standard statistical methods were utilised to assess differences between children with and without HIV infection. Descriptive analyses included variable description and cross tabulation, and inferential analyses included tests of association by chi-square test. Multivariate logistic regression analysis was done to identify factors associated with risk of MTCT and results presented as adjusted odds ratios (AOR) with 95% confidence intervals (CI). Potential confounding factors associated with the outcome and exposure (*p* < 0.10) were included in the initial multivariate logistic regression models using backward-fitting. Variables with *p* < 0.05 were used in the final model, and those that did not markedly alter the model fit were removed.

### Ethics approval

The study was approved by the University of the Witwatersrand’s Human Research Ethics Committee, and also by the Gauteng Provincial Department of Health. Written informed consent was obtained from study participants.

## Results

### Demographics and antenatal parameters

In total, 77 women with HIV-infected infants (cases), and 154 mothers with HIV-uninfected infants (controls) were interviewed. Mean age for the cases was 28.1 years (standard deviation (SD) = 6.4), and for the controls 30.9 years (SD = 5.5), *p* = 0.001; (Table 2). The proportion of participants with under 12 years of formal education was 68.8% for the cases and 44.8% for the controls, *p* <0.001. Median parity and gravidity were two for both groups. Among women with HIV-infected infants, 83.1% (64/77) had unplanned pregnancies, compared with 56.5% (87/154) of women with uninfected infants, *p* < 0.001. Median gestational age at first antenatal visit was 16.0 weeks (interquartile range (IQR) =12 – 20) for controls, and 24.0 weeks (IQR = 16 – 26) for cases, *p* < 0.001. The proportion of women who did not access any antenatal care was 1.9% (3/154) for controls and 14.3% (11/77) for cases. Of the women who did not access antenatal care and had an HIV-infected infant, six knew that they were HIV-infected. Most reasons for not accessing antenatal care revolved around fear of stigmatisation by either health workers or family.

**Table 2:** Demographics and antenatal parameters of cases and controls

**Characteristics**	**Cases (n = 77)**	**Controls (n = 154)**	***P*-value**
Age, mean (SD)	28.1 (6.4)	30.9 (5.5)	0.001
Parity, median (IQR)	2 (1 – 5)	2 (1 – 7)	0.514
Gravidity, median (IQR)	2 (1 – 6)	2 (1 – 7)	0.054
Formal education, n (%)			
≤11 years	53 (68.8)	69 (44.8)	<0.001
Knew HIV status prior to index pregnancy, n (%)	10 (13.0)	67 (43.5)	<0.001
Knew partner’s HIV status, n (%)	37 (48.1)	87 (56.5)	0.230
Unplanned pregnancy, n (%)	64 (83.1)	87 (56.5)	<0.001
Gestational age at 1^st^ antenatal visit, weeks, n (%)			
≤20 weeks	32 (45.5)	115 (78.2)	<0.001
*Baseline CD4 count (cells/mm^2^), n (%)			
≤350	34 (58.6)	54 (48.7)	0.220
Antenatal antiretrovirals received, n (%)			
AZT	48 (62.3)	83 (53.9)	<0.001
ART	9 (11.7)	66 (42.9)	
None	17 (22.9)	1 (0.7)	
Unknown	3 (3.9)	4 (2.6 )	
Intrapartum antiretrovirals received, n (%)			
sdNVP	7 (9.1)	35 (22.7)	<0.001
sdNVP and AZT	43 (55.8)	15 (9.7)	
AZT only	13 (16.9)	36 (23.4)	
ART	8 (10.4)	57 (37.0)	
None	6 (7.8)	11 (7.1)	

*CD4 cell count known for 75.3% of cases (n = 58) 72.1% of controls (n = 111).

### History of HIV infection

Of women with uninfected infants, 43.5% (67/154) knew that they were HIV-infected prior to the index pregnancy compared to 13.0% (10/77) of women with infected infants, *p* < 0.001. Slightly more than half of the women with HIV-uninfected infants knew their partner’s HIV status (56.5%; 87/154), and of the known, three-quarters were HIV-infected (73.6%), while in the group with infected infants, 37/77 (48.1%) knew their partner’s HIV status, and 94.6% were HIV-infected. Median baseline CD4 cell count was 315 cells/mm^3^ (IQR: 203 – 420) for the cases (n = 58), and 355 cells/mm^3^ (IQR: 210 – 480) for the controls (n = 111). Of women with HIV-infected infants, 22.9% (17/74) received no antenatal antiretroviral prophylaxis or treatment, while in the group with uninfected infants only one reported no antenatal antiretrovirals. In the cases, 11.7% (9/77) of the women received antenatal ART, while 42.9% (66/154) received ART in the control group.

### Intrapartum and postpartum management

A proportion of women in both groups reported receiving no intrapartum antiretrovirals – 11 (7.1%) in the group with HIV-uninfected infants, and 6 (7.8%) in the group with infected infants. Fewer women with infected infants (68/77; 88.3%) than with uninfected infants elected to formula feed (147/154, 95.4%; *p* = 0.044). In the control group, 147/148 (99.3%) infants received prophylaxis, while 72/76 (94.7%) of the cases did. The mean infant age at first HIV test was 6.0 weeks (SD = 0.2) for the uninfected infants, and 6.6 weeks (SD = 3.5) for the infected infants, *p* = 0.200. At time of interview, 73/77 (94.8%) of the infected infants had already been initiated on ART, and the mean age at ART initiation was 10.8 weeks (SD = 4.4; Table 3).

**Table 3:** Infant delivery and HIV diagnosis details

**Variable**	**Cases (n = 77)**	**Controls (n = 154)**	***P*-value**
Mode of delivery, n (%)			
NVD	48 (62.3)	106 (68.8)	0.690
C-section	29 (37.7)	46 (29.9)	
Assisted delivery	0 (0.0)	2 (1.3)	
^¥^Birth weight (g), median (IQR)	2850 (2450 – 3100)	2950 (2600 – 3200)	0.122
Infant antiretroviral prophylaxis, n (%)			
Prophylaxis received*	72 (94.7)	147 (99.3)	0.030
Infant feeding method, n (%)			
Breastfeeding	9 (11.7)	7 (4.6)	0.044
Formula feeding	68 (88.3)	147 (95.4)	
Age at 1^st^ HIV test, mean weeks; (SD)	6.6 (3.5)	6.0 (0.2)	0.200
Infant age at HIV-positive diagnosis, mean weeks (SD)	9.0 (4.2)	---	---
Infant age at ART initiation, mean weeks (SD)	10.8 (4.4)	---	---

NVD normal vaginal delivery; C-section caesarean section.

*Prophylaxis received: AZT syrup or NVP syrup; n = 76 for cases and n = 148 for controls.

^¥^Birth weight, n = 158 for controls – 4 sets of twins.

### Risk factors for MTCT

In multivariate analysis, unknown HIV status prior to conception, unplanned pregnancy, accessing antenatal care after 20 weeks gestation, and less than 12 years of formal education were all identified as important risk factors for MTCT (Table 4). The odds were 6.6 times more with an unknown HIV status, while there was a 4.3 fold increase in women presenting for antenatal care beyond 20 weeks gestation. HIV transmission was 3.4 times more likely among women with less than 12 years of formal education than those with more education (AOR 95% CI = 1.6 – 7.5), and with unplanned pregnancies it was 2.7 fold higher (AOR 95% CI = 1.2 – 6.3).

**Table 4:** Multivariate analysis of risk factors for MTCT

**Factors**	**Adjusted odds ratio AOR (95% CI)**	***P-*** **value**
Unplanned pregnancy	2.7 (1.2 – 6.3)	0.022
≤ 11 years of formal education	3.4 (1.6 – 7.5)	0.002
First antenatal visit after 20 weeks	4.3 (2.0 – 9.3)	<0.001
Unknown HIV status prior to index pregnancy	6.6 (2.4 – 18.4)	<0.001

## Discussion

This study identified several patient groups at high risk of MTCT. These were patients with unknown HIV status prior to conception; those with unplanned pregnancies; accessing antenatal care after 20 weeks gestation; and having less than 12 years of formal education. Slightly more mothers of HIV-infected infants elected to breastfeed – 11.7% compared to 4.6% of mothers with –uninfected infants, but this difference was not significant. While causality may not be definitively ascertained from this study, the findings highlight several important risk factors that must be targeted for eMTCT; availability of antiretrovirals alone is not enough.

Of women with HIV-infected infants, only 13.0% knew that they were HIV-infected prior to conception. The lowest risk of MTCT is with preconception ART and the duration of ART is directly related to MTCT risk [14,15]. HIV-infected women who initiate ART prior to pregnancy are more likely to be virally suppressed at the time of delivery – the most important determinant of MTCT. Even if ART is not initiated prior to pregnancy, there is an increased likelihood of women with known HIV status accessing PMTCT care [16]. For those women who initiate ART during pregnancy, earlier initiation of treatment is associated with a decreased risk of MTCT, and each additional week of treatment confers a cumulative reduction in risk [15,17,18].

If we are to eliminate MTCT, there needs to be routine HIV testing for all women and men of reproductive age, and ART initiation for those found to be HIV-infected and eligible for treatment. South Africa implemented large population-based HIV-testing campaigns in recent years, but it is uncertain if these have impacted on PMTCT and linkage to care. However, recent unpublished programmatic data from the Soweto PMTCT programme, where this study was conducted, shows an increasing proportion of pregnant women presenting with an already known HIV status, and an increasing number already on treatment. As HIV testing and treatment programmes expand and become functional, more women will know their HIV status preconception. They will however still need to present for antenatal care timeously for early initiation and continuation of appropriate PMTCT interventions. Healthcare services need to be able to manage the increased demand for both HIV testing and initiation of ART in those found to be eligible.

One area that is under-researched, but increasingly shown to be important in the success of PMTCT interventions, is male partner involvement [19-21]. Encouraging male partners to be involved in antenatal care, including routine HIV testing for themselves, has been shown to increase the likelihood of HIV-infected pregnant women accessing and adhering to treatment [19,20]. More importantly, it has been linked with a decreased risk of MTCT, and increased HIV-free survival in HIV-uninfected infants [21]. In our study, less than half of the women with infected infants knew their partners’ HIV status, and of the known, the majority were HIV-infected; the proportions were marginally better for women with HIV-uninfected infants, but still suboptimal. There are several barriers to male partner involvement in PMTCT programmes, and more broadly in sexual and reproductive health services. Some of the most pertinent barriers identified include the cultural perception, and often societal norm, that men do not participate in reproductive health services and that the facilities where these are offered are not male-friendly [22]. Various strategies have been utilised to encourage more male partner participation, and amongst them, healthcare provider-driven initiatives to invite men to be part of reproductive and PMTCT programmes, and community-based strategies which include male peer support groups and community campaigns [22,23].

Rates of unplanned pregnancies were high, especially amongst women who had infected infants – 83.1% of the pregnancies were unplanned. Family planning, and consequently prevention of unintended pregnancies, has long been the most underutilised PMTCT intervention, with only modest progress to date [24]. In addition to decreased risk of MTCT, planned pregnancies are also associated with better obstetric outcomes, with decreased maternal morbidity and mortality [24]. There remains a high unmet need for contraception among HIV-infected women, and even in those who have access to reproductive health services, rates of unplanned pregnancies remain high [25,26]. Integration of reproductive health and PMTCT is one of the suggested strategies for improving the reproductive health outcomes of HIV-infected women [27]. There is however a need for stronger programmatic efforts and implementation research for integration to work [27].

Timing of antenatal care influences diagnosis and appropriate management of maternal HIV infection. In our study, accessing antenatal care beyond 20 weeks gestation was associated with an increased MTCT risk. This is consistent with findings from other studies where late presentation for antenatal care delayed initiation of appropriate PMTCT interventions, increasing the risk of perinatal transmission [16,17]. Lack of integration of antenatal and HIV services also contributes to the delay in HIV-infected women initiating treatment [28]. There are several reasons why pregnant women either do not access antenatal care, or delay doing so, and these include lack of access to care; poor knowledge about timing and need to present for care; and unavailability of services [29]. It is also important that once HIV-infected women access antenatal care, they remain in care. While adherence to ART has been shown to be higher prenatally than postpartum, there is still significant loss to follow-up in the PMTCT continuum of care, and patient and health system factors have been identified as contributing factors [16]. There is a need for targeted interventions, both prenatally and postpartum, to ensure that HIV-infected women remain in care.

While there is increased public knowledge of HIV infection, stigma remains a reality for many, especially women. In our study, there were women who knew that they were HIV-infected, but still did not access antenatal care due to fear of being stigmatised by either health workers or family. There’s a need to address stigma at all levels, as it has been shown to impact health-seeking behaviour and adherence to treatment [30]. Patient information on PMTCT interventions should be simplified and reinforced at every opportunity; understanding and adherence to treatment need not depend on level of education. A low level of formal education was one of the risk factors for MTCT identified. Inadequate knowledge of PMTCT interventions among health workers was also highlighted in a cross-sectional survey assessing quality of care within the same Soweto programme as this study [31].

Despite the challenges identified, the study provides several reassuring findings. Routine early infant diagnosis, and ART initiation in those found to be HIV-infected were timeous. At the time of the study, 94.8% of the HIV-infected infants had already been initiated on ART, and the mean age at ART initiation was 10.8 weeks.

While this study makes an important contribution to our knowledge of the requirements for an effective PMTCT programme, and highlights the importance of patient factors as we work towards elimination of MTCT, there are limitations. Causality cannot be inferred between the negative patient factors identified and risk of MTCT. There was also a lag period between the interviews of cases and that of controls, and this could have contributed to recall bias amongst the controls as they were interviewed at a much later period post-delivery. The sample of controls might also not have been representative as there was a high percentage that could not be reached, and some of the infants might have died during the lag period as it is a recognised fact that despite being uninfected, the morbidity and mortality rate in HIV-exposed infants remains significant. We also relied on self-reported information, but this was found to be reliable when compared to hospital records.

## Conclusions

Despite the limitations, the study provides important insights as we work towards eMTCT. As much as availability and implementation of efficacious PMTCT interventions are key in preventing HIV infection in children, individual patient factors are also important and need to be targeted. Undiagnosed maternal HIV infection prior to conception, unplanned pregnancies, delays in accessing antenatal care, and low levels of education were the most significant patient risk factors associated with MTCT in our study. The focus in resource-constrained settings has largely been on establishing functioning systems to facilitate implementation of efficacious PMTCT interventions, and great strides have been made in this regard, including in South Africa [24,32]. While continued strengthening of health systems is crucial, our study highlights the considerable importance of individual patient factors for further reducing paediatric HIV infections. There is a need for further research on why rates of unplanned pregnancies, and consequently considerable delays in accessing antenatal care, remain high. Also, why the rates of preconception HIV testing remain low in resource-constrained settings, despite the availability of interventions to address these factors.

## Abbreviations

AIDS: Acquired Immune Deficiency Syndrome; AOR: Adjusted odd ratio; ART: Antiretroviral therapy; AZT: Zidovudine; CI: Confidence intervals; C-section: Caesarean section; EFV: Efavirenz; eMTCT: Elimination of mother-to-child transmission of HIV; FDCs: Fixed dose combinations; HIV: Human Immunodeficiency Syndrome; IQR: Interquartile range; MTCT: Mother-to-child transmission of HIV; NVD: Normal vaginal delivery; NVP: Nevirapine; PMTCT: Prevention of mother-to-child transmission of HIV; SD: Standard deviation; sdNVP: single-dose nevirapine; UNAIDS: Joint United Nations Programme on HIV/AIDS; WHO: World Health Organization.

## Competing interests

We declare that we have no conflicts of interest.

## Authors’ contributions

CNM conceptualised the study and wrote the manuscript. AS performed the statistical analysis. MC helped to draft the manuscript. JM assisted with the statistical analysis. JAM participated in the study design and helped to draft the manuscript. All authors read and approved the final manuscript.
